# Multiple functions of exogenous melatonin in cucumber seed germination, seedling establishment, and alkali stress resistance

**DOI:** 10.1186/s12870-025-06359-3

**Published:** 2025-03-19

**Authors:** Qiuxia Li, Yiqiu Zhang, Yu Liu, Tianyue Li, Hua Xu, Qinwen Wei, Huiliang Zeng, Huiyi Ni, Shuzhen Li

**Affiliations:** Ganzhou Key Laboratory of Greenhouse Vegetable, College of Life Science, Gannan Normal University, Ganzhou, 341000 China

**Keywords:** Melatonin, Cucumber, Seed germination, Seedling growth, Alkali stress

## Abstract

**Background:**

Exogenous melatonin plays a crucial role in various plant developmental processes and stress responses and has considerable potential for future agricultural applications. However, its effects on early cucumber seedling growth and resistance to alkaline stress have not been adequately explored. This study investigated the role of exogenous melatonin during the early growth stages of cucumber, specifically focusing on seed germination, post-germination seedling growth, and 1-leaf stage seedling growth, with particular emphasis on its influence on alkali stress resistance. These findings are intended to enhance the application of melatonin in cucumber seedling cultivation and provide a theoretical basis for promoting growth and improving stress tolerance in agricultural production.

**Results:**

Exogenous melatonin enhanced cucumber seed germination and early seedling growth with promoting and inhibitory effects at low and high concentrations, respectively. However, the effects of exogenous melatonin on cucumber growth varied at different developmental stages. Additionally, alkali stress significantly hampered the growth of cucumber seedlings; however, the external application of melatonin mitigated the damage caused by this stress. This protective effect was evidenced by a marked increase in the survival rate, stem diameter, and biomass of cucumber seedlings, along with a significant reduction in malondialdehyde content and electrolyte leakage rate. Further investigation revealed that exogenous melatonin promotes the accumulation of osmoregulatory substances, specifically soluble sugars, and proline, under alkaline stress. It also enhances the activities of antioxidant enzymes, including peroxidase, superoxide dismutase, catalase, and dehydroascorbate reductase, while significantly decreasing the accumulation of reactive oxygen species such as H_2_O_2_ and O_2_⋅−. Furthermore, exogenous melatonin increased the activities of PM-H^+^-ATPase and V-H^+^-ATPase and stimulated the expression of stress-related genes, thereby regulating Na^+^ and K^+^ homeostasis under alkali stress. Additionally, exogenous melatonin promoted the synthesis of endogenous melatonin in cucumbers subjected to alkaline stress by inducing the expression of melatonin synthase genes, namely, *CsASMT*, *CsCOMT*, *CsTDC*, and *CsSNAT*.

**Conclusions:**

Exogenous melatonin promoted cucumber seed germination and seedling establishment and enhanced cucumber alkali stress tolerance by mediating osmotic adjustment, reactive oxygen species scavenging, ion homeostasis maintenance, endogenous melatonin synthesis, and expression of stress-related genes.

**Supplementary Information:**

The online version contains supplementary material available at 10.1186/s12870-025-06359-3.

## Introduction

Global climate change has exposed higher plants to adverse environmental stressors, including extreme temperatures, high salinity, and drought [1]. Salinity stress has become a significant global issue due to improper soil management practices, severely impacting the growth and productivity of key crops in arid and semiarid regions [2]. By 2050, an estimated 50% of the world’s arable land is expected to experience salinization [3]. Salinity stress can be categorized into two main types: neutral salt stress, commonly referred to as salt stress, which is primarily caused by Na_2_SO_4_ and NaCl, and alkaline salt stress, often termed alkali stress, which is mainly induced by NaHCO_3_ and Na_2_CO_3_ [4–6]. Both salt and alkali stress induce oxidative stress, ion toxicity, and osmotic stress in plants, however, alkali stress poses additional harm to plants because of elevated rhizosphere soil pH levels [7, 8]. This stress not only impairs root uptake capacity, but also diminishes nutrient availability and disrupts ionic balance [9, 10]. Consequently, alkali stress poses a greater threat to plants than salt stress.

The plasma membrane (PM) H^+^-ATPase facilitates the transport of protons from the cytoplasm to the apoplast through the hydrolysis of ATP, thereby playing a crucial role in maintaining the pH balance both inside and outside the cell [11]. Furthermore, the regulatory network of salt-alkali stress signals mediated by PM-H^+^-ATPase represents a significant molecular mechanism by which plants respond to salt-alkali stress [12]. A high concentration of Na^+^ is another main cause of alkali stress in plants, as it can induce ion toxicity due to the similar ionic radii with K^+^. This similarity inhibits K^+^ uptake, leading to an excessive accumulation of Na^+^ in the shoots [13]. Notably, shoots exhibited greater sensitivity to salt-alkali stress than roots, with the phenotypic symptoms of salt damage appearing earlier [14]. Consequently, expelling excess Na^+^ from the plant or compartmentalizing it in the vacuole is a crucial strategy for maintaining ion homeostasis and enabling plants to withstand salt-alkali stress, which involves the participation of numerous genes [15]. The plasma membrane Na^+^/H^+^ antiporter SOS1 facilitates Na^+^ efflux, whereas the tonoplast Na^+^/H^+^ antiporter NHX1 is responsible for Na^+^ compartmentalization. Both processes rely on proton gradients generated by PM-H^+^-ATPase and vacuolar membrane (V) H^+^-ATPase [15]. The V-type proton ATPase proteolipid subunit (VHA) encodes a subunit of the V-H^+^-ATPase [16]. In addition, SOS2, a serine/threonine protein kinase (CIPK), interacts with SOS3, a calcium-binding protein (CBL), to form a kinase complex that subsequently activates SOS1, thereby facilitating the expulsion of Na^+^ from the cell [15]. The high-affinity K^+^ transporter (HKT) enhances K^+^ absorption during salt-alkali stress and is essential for maintaining the balance of Na^+^ and K^+^ [17, 18]. Consequently, during K^+^ deficiency, the expression of *HKT* is upregulated [19]. Additionally, the K^+^ efflux antiporter (KEA) is recognized as a K^+^-selective uptake protein that regulates intracellular K^+^ levels and pH homeostasis, thereby contributing to plant tolerance to salt-alkali stress [20]. Furthermore, alkali stress leads to the precipitation of Ca^2+^ in the soil, which impedes the absorption of Ca^2+^ by plants. CAX, a Ca^2+^/H^+^ exchanger antiporter, plays a crucial role in regulating Ca^2+^ homeostasis and is a direct target of SOS2 [21]. Although there is a comprehensive understanding of plant salt tolerance, knowledge of plant tolerance to alkali stress remains limited. It is crucial to explore effective strategies for enhancing plant tolerance to alkaline stress, a topic requiring significant attention from the scientific community.

Melatonin (N-acetyl-5-methoxytryptamine, MT) is a small indoleamine molecule that is widely present in both animals and plants [22]. The discovery of the MT receptor (CAND2/PMTR) in *Arabidopsis thaliana* suggested that MT may function as a plant hormone [23], and the term “phytomelatonin” was proposed subsequently [24]. There are four primary pathways for the synthesis of phytomelatonin, each utilizing tryptophan as a substrate and involving the participation of at least six enzymes: tryptophan decarboxylase (TDC), N-acetylserotonin methyltransferase (ASMT), caffeic-O-methyltransferase (COMT), tryptamine 5-hydroxylase (T5H), tryptophan hydroxylase (TPH), and serotonin N-acetyltransferase (SNAT) [25]. Since 1995, when MT was first identified in plants, extensive research has been conducted on phytomelatonin [26, 27]. Numerous studies have demonstrated that MT serves as an effective plant growth regulator, playing a crucial role in various aspects of plant development, such as seed germination [28, 29], stem strength enhancement [30], root formation [31], leaf senescence [32], flower development, and fruit ripening [33]. The lower concentrations of MT (50 or 100 µM) promoted soybean seed germination, and seedlings from MT-coated seeds exhibited larger leaves and higher height [34]. MT also promoted the growth of aboveground parts of stevia plantlets in a concentration-dependent manner, including fresh weight, leaf numbers, and stem length [35]. Besides, MT shares structural similarities with indoleacetic acid (IAA) and utilizes the same synthetic substrate (tryptophan), and is therefore thought to have auxin-like functions, including the induction of stem and root growth, as well as the stimulation of lateral root production [36]. Yang et al. [31] demonstrated that MT promotes the growth of the primary root in Arabidopsis in an IAA-dependent manner. MT is also an efficient free radical scavenger and antioxidant, that can directly scavenge excess reactive oxygen species (ROS), and enhance the activities of antioxidant enzymes such as superoxide dismutase (SOD), peroxidase (POD), and catalase (CAT) [37], thereby improving plant tolerance to various abiotic stresses, including drought [38, 39], salt [40, 41], high temperature [42], low temperature [43], nitrogen deficiency [44], and copper toxicity [45]. Although some studies have indicated that MT may improve the alkali stress tolerance of horticultural plants [46], there is a notable scarcity of research on this topic, and the specific effects of MT on the regulation of alkali stress resistance in cucumbers remain unclear. The pleiotropic effect of MT makes it a promising regulator of agricultural production, with the potential to decrease reliance on fertilizers and pesticides; thus, exploring its function in horticultural plants has emerged as a rapidly developing field [47].

Cucumber (*Cucumis sativus* L.) is an important cash crop cultivated worldwide. The seed germination and establishment of early seedlings are critical for developing robust cucumber plants and increasing production. Besides, cucumber is highly susceptible to alkali stress, which significantly hinders its sustainable production [48]. Therefore, it is imperative to implement effective strategies to promote the growth of early cucumber seedlings and enhance cucumber tolerance to alkali stress. In this study, we examined the effects of exogenous MT on cucumber seed germination and seedling growth. Subsequently, a concentration of 5 µM MT was utilized to explore the mechanisms by which MT mitigates alkali stress, including osmoregulation, scavenging of ROS, maintenance of ion homeostasis, modulation of MT synthesis pathways, and regulation of alkali stress-related gene expression. These results provide a theoretical basis for MT application to promote cucumber seedling growth and increase alkali resistance.

## Materials and methods

### Seed germination analysis

Cucumber seeds (‘Xinjinyan No. 4’, obtained commercially from Shandong Tai’an Huayi Seed Industry Co., Ltd., China) were soaked in water at 55 °C for 30 min. Subsequently, the seeds were placed in a petri dish with 8 mL of MT (McLean, Shanghai, China) solution at varying concentrations (0, 1, 5, 10, 20, 50, 100, 200, and 400 µM). Each petri dish (9 cm in diameter and lined with two layers of filter paper) contained 50 seeds, and each treatment was replicated three times. The MT was dissolved with small amounts of dimethyl sulfoxide (DMSO) (McLean, Shanghai, China), and equal volume of DMSO was added to all experiment groups. Cucumber seeds were deemed to have germinated once their seed coat fractured and the radicle appeared white. Germination rate was assessed at 10, 12, 14, 16, 18, 20, 22, and 24-hours post-treatment, with subsequent measurements of radicle length and fresh weight of germinated cucumber seeds at 36 h.

### Plant growth and alkali stress treatment

Cucumber seeds were germinated and planted in 32-hole pots filled with cultivation substrate. Once the cotyledons had unfolded, seedlings displaying consistent growth were transplanted into polyvinyl chloride pots (33 × 25 × 11 cm) containing 5 L 1/4 Hoagland nutrient solution [49], with 12 plants in each pot. Following the emergence of the first true leaf, treatments with MT at varying concentrations (0, 1, 5, 10, 20, 50, 100, 150, and 200 µM) were administered, with each treatment being replicated three times. During this period, the seedlings were cultured in a 1/4 Hoagland nutrient solution until the first true leaf reached half-expanded. Subsequently, they were transferred to a 1/2 Hoagland nutrient solution. On the 7th day of treatment, growth indicators were measured to determine the optimal concentration of MT (5 µM/L).

For the alkali stress treatment (Refer to Supplementary Fig. 1 for the alkali concentration screening), cucumber seedlings were cultivated as described above. Once the first true leaf had expanded, the seedlings were divided into four groups, each containing six pots. The four treatment groups were as follows: (1) CK group: 0 mM/L NaHCO_3_ + 0 µM/L MT, (2) MT group: 0 mM/L NaHCO_3_ + 5 µM/L MT, (3) SB group: 50 mM/L NaHCO_3_ + 0 µM/L MT, (4) SB-MT group: 50 mM/L NaHCO_3_ + 5 µM/L MT. The seedlings were cultured in 1/2 Hoagland nutrient solution, which had an initial pH of 6.23. After the addition of 50 mM NaHCO₃, the pH of the nutrient solution increased to 7.07. Seedlings in the SB-MT group were pre-treated with MT one day prior to the alkali stress treatment. Cucumber leaves were collected on the 4th and 8th days of the treatment for physical and chemical index measurements. Gene expression analysis was conducted on leaves collected on the 4th day. Survival rate, stem diameter, and plant biomass were measured on the 8th day, and the stem lodging was used as the criterion for seedling death.

Cucumber seedlings were cultivated and treated in the artificial climate chamber at the School of Life Sciences, Gannan Normal University (25 ± 1 °C, 12-h light/12-h dark cycle). The nutrient solution was refreshed every 2 d before and after treatment.

### Growth index determination

Seedling lateral root number and leaf area were determined by analyzing 2D images of the roots and leaves using Wanshen LA-S plant scanning and analysis systems (Wanshen Testing Technology Co., Ltd., Hangzhou, China). Plant height (from the base of the stem to the growing point) and root length were measured using a ruler, whereas stem diameter (stem base) was measured using a Vernier caliper. Deionized water was used to clean the plants, and surface moisture was wiped off. The fresh weights of both the aboveground and roots of the seedlings were measured. Subsequently, the plants were placed in an oven and dried at 105 °C for 15 min, followed by drying at 55 °C to a constant weight. The dry weights of both shoots and roots were then measured.

### Malondialdehyde, electrolyte leakage, soluble sugar and proline content assays

Malondialdehyde (MDA), electrolyte leakage, and soluble sugar and proline content were determined as our previously described [13].

MDA content: Weigh 0.3 g of the fresh sample and add 3 mL of phosphate buffer (50 mmol/L, pH 7.8). Grind the sample into a homogenate, then centrifuge at 4 °C at 12,000 rpm for 20 min. Take 1 mL of the supernatant (1 ml of distilled water for the control). Next, add 2 mL of 0.67% thiobarbituric acid (TBA) and boil the mixture in a water bath for 15 min. Afterward, cool the mixture to room temperature, then centrifuge again at 12,000 rpm for 20 min, the supernatant was assayed at 600 nm, 532 nm, and 450 nm.

Electrolyte leakage: Use a hole punch to obtain a 0.1 g leaf disk sample. Place the sample in a glass test tube containing 10 mL of distilled water and incubate it at 32 °C for 2 h. After this period, measure the initial conductivity (EC1). Next, transfer the test tube to a boiling water bath for 20 min, then allow it to cool to 25 °C before measuring the final conductivity (EC2). Additionally, measure the conductivity of the distilled water (EC3) simultaneously. The electrolyte leakage rate (EL) can be calculated using the formula: EL = (EC1 − EC3) / (EC2 − EC3) × 100%.

Soluble sugar content: Weigh 0.03 g of the dried and ground sample and place it into a glass test tube. Add 10 mL of distilled water and boil in a water bath for 30 min, repeating this process twice. Next, filter the mixture using filter paper and dilute the resultant filtrate to a final volume of 50 mL. Take 1 mL of the filtrate and add 1 mL of distilled water. For the control, add 2 mL of distilled water directly. Subsequently, add 0.5 mL of anthrone ethyl acetate, followed by 5 mL of concentrated H_2_SO_4_. Shake and mix the solution, then place it in a boiling water bath for 1 min. Afterward, cool the mixture to room temperature and measure the absorbance at 630 nm.

Proline content: Weigh 0.03 g of the dried and ground sample into a glass test tube. Add 5 mL of 3% (w/v) sulfosalicylic acid and boil the mixture in a water bath for 10 min. After this period, filter the mixture using filter paper and collect 2 mL of the filtrate. For the control, use 2 mL of distilled water instead. Next, add 2 mL of glacial acetic acid and 3 mL of acidic ninhydrin hydrate chromogenic solution. Boil this mixture in a water bath for an additional 40 min. Once cooled, add 5 mL of toluene, shake the mixture thoroughly, and allow it to stand so that the layers can separate. Finally, collect the upper layer of the toluene liquid and measure the absorbance at 520 nm.

### Antioxidant enzyme activities, O2.− production rate, and H2O2 content assays

The hydrogen peroxide (H_2_O_2_) content was determined using the titanium oxidation method described by Patterson et al. [50]. The O_2_.^−^ production rates and activities of the antioxidant enzymes superoxide dismutase (SOD), peroxidase (POD), and catalase (CAT) were measured as described by Tian et al. [51]. Ascorbate peroxidase (APX) and dehydroascorbate reductase (DHAR) activities were detected as described by Nakano and Asada [52]. The glutathione reductase (GR) activity was detected using a kit (Suzhou Keming Biotechnology Co., Ltd., Suzhou, China).

### Determination of Na+ and K+ contents

Inductively coupled plasma mass spectrometry (ICP-MS) was used to analyze Na^+^ and K^+^ contents. Initially, the collected samples were dried at 105 °C for 15 min, followed by drying at 55 °C to a constant weight. The dried samples were then ground in a mortar and passed through a 100-mesh nylon sieve (pore diameter: 0.149 mm). A 0.1 g sample was weighed into a 30 mL polytetrafluoroethylene crucible and 8 mL of a mixed acid solution consisting of concentrated nitric acid, hydrofluoric acid, and perchloric acid in a 3:3:1 ratio. The mixture was heated at 250 °C until the perchloric acid is completely exhausted, indicated by the sample being evaporated. The heat source was removed and the temperature was allowed to decrease to around 180 °C. Then, 8 mL of aqua regia and 10 mL of an internal standard mixture were added, mixed thoroughly, and allowed to sit overnight for ICP operation. The following day, 250 µL of the digested solution were taken, 5 mL of 3% nitric acid solution added, mixed well, and used for ICP-MS analysis using a PerkinElmer NexION 300D (PerkinElmer, Waltham, MA, USA).

### Determination of PM-H+-ATPase and V-H+-ATPase activity

Approximately 0.1 g of cucumber leaves were collected and ground into powder using a grinder. Subsequently, 0.9 mL of phosphate buffer (0.05 mol/L, pH 7.4) was added, mixed thoroughly, and the mixture was centrifuged at 4000 rpm for 20 min at 4 °C. The supernatant was collected and plant PM-H^+^-ATPase and V-H^+^-ATPase ELISA kits (Jiangsu Meimian Industrial Co., Ltd., China) were used to quantify plasma membrane PM- H^+^-ATPase and vacuolar membrane V-H^+^-ATPase activities.

### Determination of endogenous MT content

The MT content was analyzed by Shanghai Haling Biotechnology Co., Ltd. (Shanghai, China) using high-performance liquid chromatography (HPLC). Cucumber leaves (0.2 g) were homogenized in 1 mL pre-cooled methanol using a tissue lyser at 60 Hz for 10 min. The mixture was then transferred to a 15 mL brown centrifuge tube, and 4 mL of pre-cooled methanol was added and thoroughly mixed on a shaker for 5 min. Subsequently, the sample was sonicated at 4 °C in the dark for 60 min, followed by centrifugation at 4000 rpm for 10 min. The supernatant was carefully transferred to a new 15 mL brown centrifuge tube, and 2 mL of pre-cooled methanol was added. The extraction process was repeated once and the two supernatants were combined and mixed by vortexing. Streaming nitrogen was used to dry the samples at room temperature, which were then reconstituted in 50% MeOH (0.5 mL). After thorough mixing, the supernatant was filtered through a 0.22 μm filter membrane for HPLC analysis (HPLC1200, Agilent Technologies, Santa Clara, CA, USA).

### qRT-PCR analysis

Total RNA from cucumber leaves was extracted using the MolPure^®^ Plant Plus RNA Kit (Yeasen, Shanghai, China), and cDNA was synthesized using Hifair^®^ III 1st Strand cDNA Synthesis SuperMix for qPCR (gDNA digester plus) (Yeasen). The qPCR reaction was performed on a LightCycler^®^ 96 Instrument (Roche, Mannheim, Germany) following the instructions of Hieff^®^ qPCR SYBR Green Master Mix (No Rox) (Yeasen). Relative gene expression was calculated using the 2^−∆∆Ct^ method [53]. The primers used for qPCR analysis are listed in Supplementary Table 1.

### Statistical analysis

Values are presented as the means ± standard deviation (SD). Significance analysis was conducted using SPSS 20.0, statistical software, and STST software by one-way analysis of variance (ANOVA) and Duncan’s test.

## Results

### Exogenous MT enhances cucumber seed germination under normal growth conditions

**Fig. 1:**
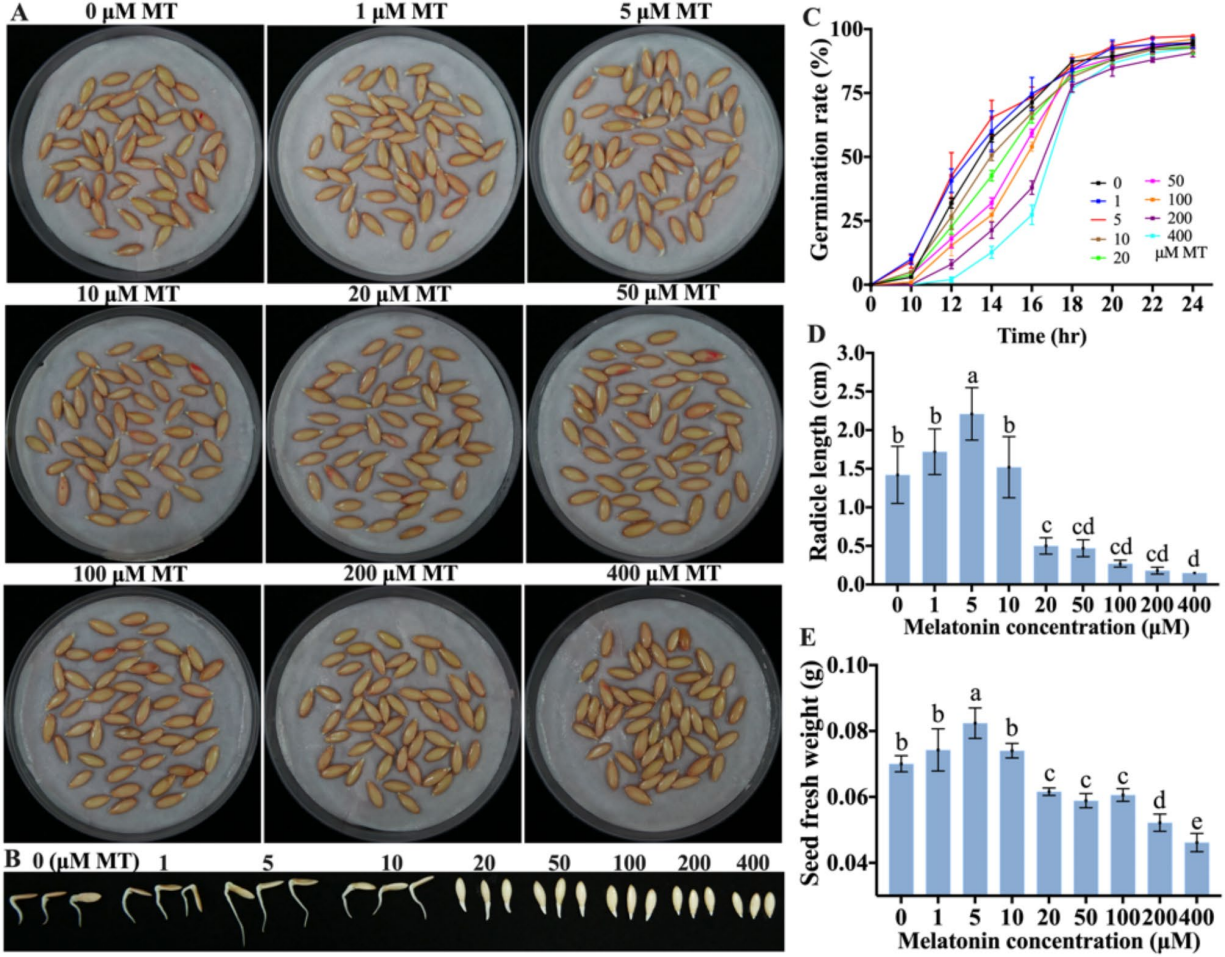
Effects of exogenous MT on cucumber seed germination under normal growth conditions. (**A**–**B**) Germination of cucumber seeds when treated with MT at different concentrations for 18 and 36 h. (**C**) Germination rate of cucumber seeds treated with different concentrations of MT. Data are presented as the mean ± SD of three independent experiments, each containing 50 seeds. (**D**) Radicle length of cucumber seeds treated with various concentrations of MT for a duration of 36 h. Values are means ± SD (*n* = 5). (**E**) Fresh weight of cucumber seeds treated with various concentrations of MT for a duration of 36 h. Values are means ± SD of three average weights from three groups, with each containing 50 seeds. Different letters indicate significant differences (*p* < 0.05)

To investigate the effects of exogenous MT on cucumber seed germination, experiments were conducted using varying MT concentrations. Figure 1C illustrates that after 10 h, seeds treated with 0 µM MT had a germination rate of 3%, whereas seeds treated with 1, 5, 10, 20, 50, 100, 200, and 400 µM MT had germination rates of 10%, 9%, 5%, 4%, 4%, 1%, 0%, and 0% respectively. Over the following 12–16 h, seeds treated with 1 and 5 µM MT exhibited higher germination rates compared to those treated with 0 µM, while seeds treated with 10 µM had lower germination rates than the control. Furthermore, there was a gradual decrease in the germination rate as MT concentration increased. After 18 h, the differences in germination rates among the different MT concentrations began to diminish. Notably, at this point radicles of seeds treated with 5 µM MT began to bend, whereas those treated with 0, 1, 10, and 50 µM MT had just penetrated the seed coat, and seeds treated with 100, 200, and 400 µM MT had just cracked the seed coat (Fig. 1A). After 24 h, the seed germination rates across treatments exceeded 90%. By 36 h, the radicle length (Fig. 1B and D) and fresh weight (Fig. 1E) of cucumber seeds treated with different MT concentrations initially increased, then decreased as the MT concentration rose. The peak values were observed at 5 µM MT treatment and declined at 20 µM, indicating an inhibitory effect. These findings highlight the concentration-dependent effect of MT on cucumber seed germination, with lower concentrations promoting germination and higher concentrations inhibiting it. Specifically, a favorable promotion effect was observed at an MT concentration of 5 µM in this study.

### Exogenous MT promotes cucumber seedling growth under normal growth conditions

**Fig. 2:**
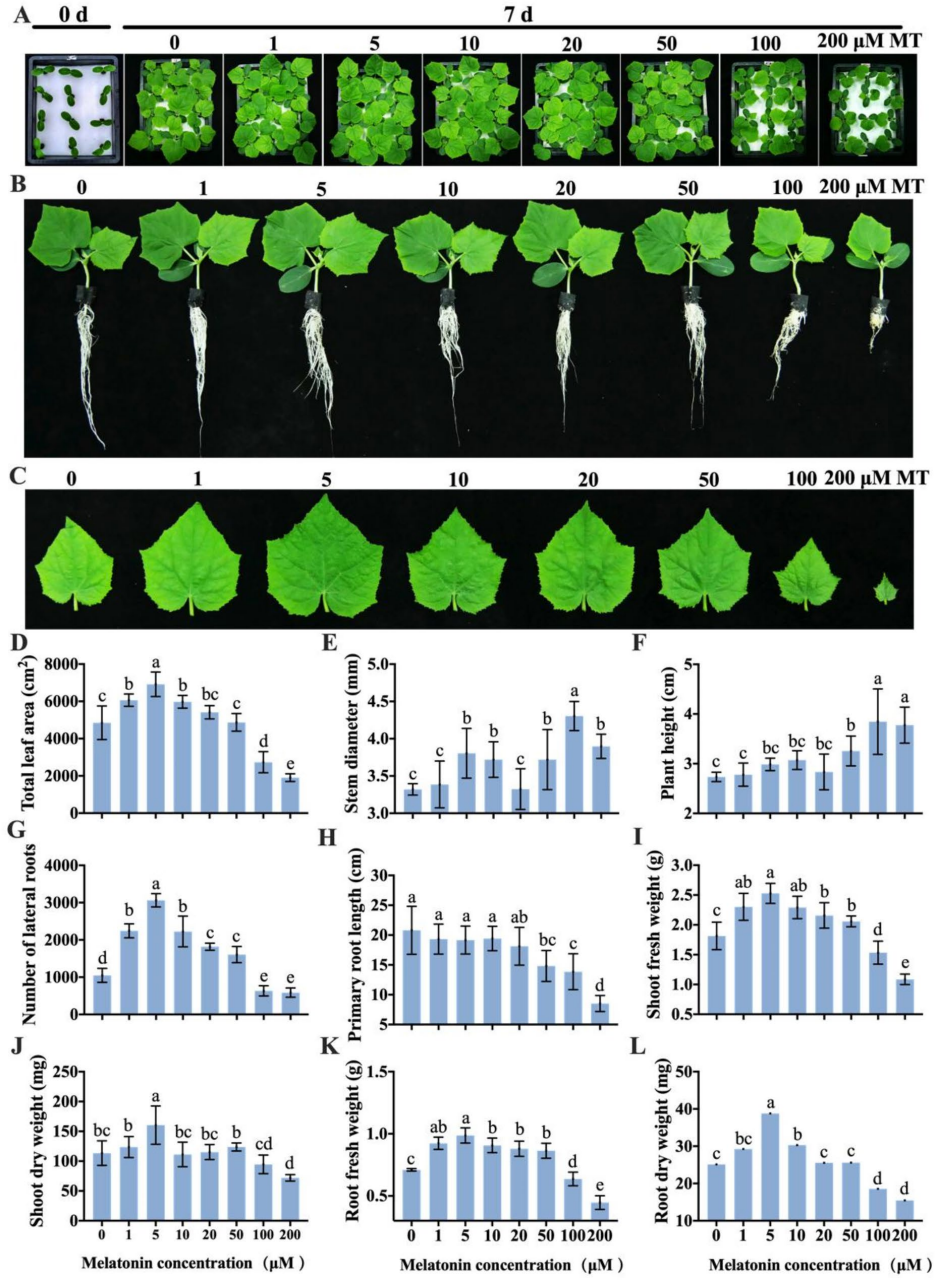
Effects of exogenous MT on cucumber seedling growth under normal growth conditions. (**A**–**C**) Phenotypes of cucumber seedlings before and 7 d after treatment with various concentrations of MT. The total leaf area (**D**), stem diameter (**E**), plant height (**F**), number of lateral roots (**G**), and primary root length (**H**) of cucumber seedlings were measured after treatment with MT for a duration of 7 d. Data are presented as the mean ± SD (*n* = 5 random independent seedlings). (**I**–**L**) Shoot and root biomass of cucumber seedlings after being treated with MT for a period of 7 d. Values are means ± SD of three average weights from three groups, with each containing 5 seedlings. Different letters indicate significant differences (*p* < 0.05)

To investigate the effect of exogenous MT on cucumber seedling growth and foster sturdy cucumber seedlings, we examined the effects of varying concentrations of MT on cucumber seedling growth indicators. The findings revealed that low concentrations of MT stimulated the growth of lateral roots (Fig. 2G), leaves (Fig. 2A, C, and D), shoots (Fig. 2I and J), and roots (Fig. 2K and L), whereas high concentrations of MT hindered growth. The most effective promotion was observed with a concentration of 5 µM, while growth was inhibited with concentrations of 100 and 200 µM. Moreover, higher concentrations (100 and 200 µM) of MT promoted the thickening (Fig. 2E) and elongation (Fig. 2F) of cucumber seedling stems, albeit with abnormal morphology, characterized by fluffy and white seedling stem bases. Furthermore, lower MT concentrations (≤ 20 µM) had no impact on cucumber seedling root elongation, whereas higher concentrations (≥ 50 µM) inhibited root elongation (Fig. 2B and H). Overall, the study determined that an appropriate concentration of MT enhanced cucumber seedling growth, with the optimal concentration identified as 5 µM.

### Exogenous MT improves the alkali stress tolerance of cucumber seedlings

**Fig. 3:**
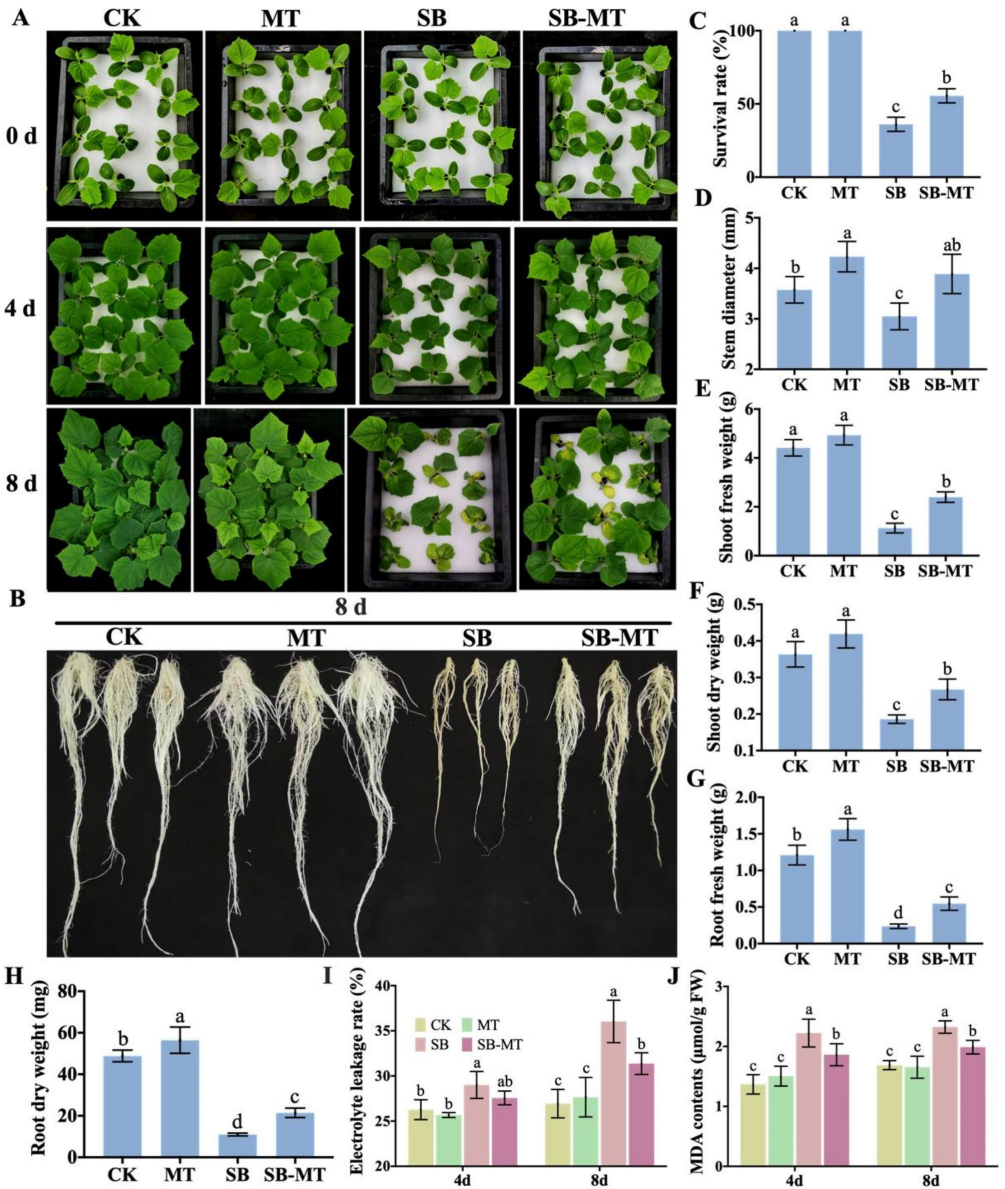
Effects of exogenous MT on cucumber seedling growth under alkali stress conditions. (**A**–**B**) The phenotypic variations of cucumber seedlings across different treatment groups exposed to alkali stress. (**C**) Survival rate of cucumber seedlings under NaHCO_3_ treatment for 8 d. Data are presented as the mean ± SD (*n* = 3, 12 seedlings per replicate). (**D**) Stem diameter of cucumber seedlings under NaHCO_3_ treatment for 8 d. Values are means ± SD (*n* = 6). (**E**–**H**) Shoot and root biomass of cucumber seedlings after being treated with NaHCO_3_ for 8 d. Values are means ± SD (*n* = 3, 5 seedlings per replicate). (**I**–**J**) The electrolytic leakage rate and MDA content. Values are means ± SD (*n* = 3). Different letters indicate significant differences (*p* < 0.05)

To further investigate the role of exogenous MT in enhancing cucumber stress resistance, particularly alkali stress, and to enhance the alkali stress tolerance of cucumber seedlings, the optimal MT concentration for cucumber seedling growth (5 µM) was selected for the subsequent alkali tolerance analysis. As depicted in Fig. 3, alkali stress had a severe inhibitory effect on the growth of cucumber seedlings, and the level of inhibition increased significantly as the duration of stress increased, ultimately resulting in plant death (Fig. 3A and B). However, the external application of MT alleviated alkali stress damage, as evidenced by notable increases in the survival rate (Fig. 3C), stem diameter (Fig. 3D), and fresh and dry weights of shoots and roots (Fig. 3E–H) of cucumber seedlings. Furthermore, under normal growth conditions, the external application of MT had no significant effect on electrolyte leakage rate (Fig. 3I) or MDA content (Fig. 3J). However, under alkali stress, MT application significantly reduced electrolyte leakage rate and MDA content in cucumber seedlings. These findings suggest that exogenous MT application improves alkali stress tolerance in cucumber seedlings.

### Exogenous MT promotes the synthesis of osmotic substances in cucumber under alkali stress

**Fig. 4:**
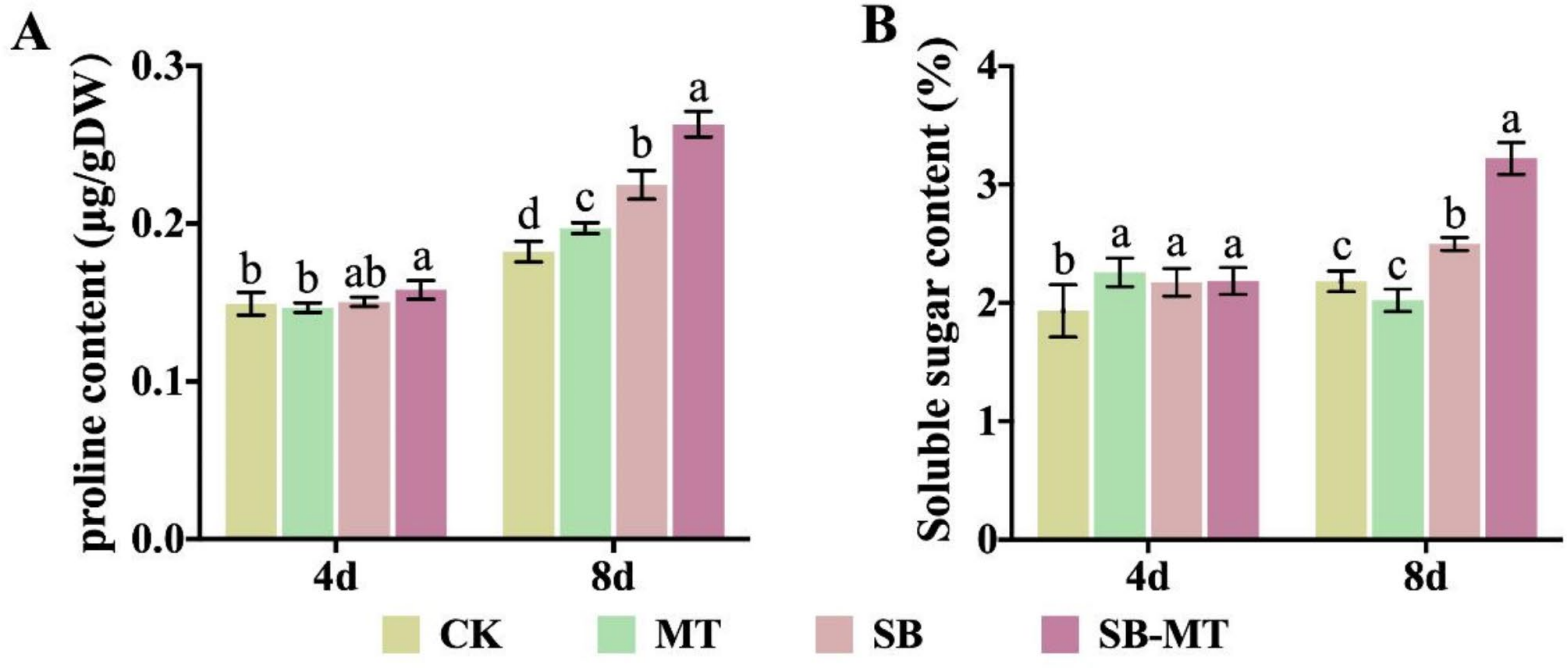
Effects of exogenous MT on osmotic substances in cucumber seedlings exposed to alkali stress. The proline (**A**) and soluble sugar contents (**B**) of cucumber seedlings were measured at 4 and 8 d following NaHCO_3_ treatment in different experimental groups. Values are means ± SD (*n* = 3). Different letters indicate significant differences (*p* < 0.05)

To investigate the effect of exogenous MT on enhancing cucumber alkali resistance, we investigated the osmotic regulation pathway and examined the levels of proline and soluble sugars. Figure 4 illustrates that exposure to alkali stress led to a swift build-up of proline (Fig. 4A) and soluble sugars (Fig. 4B) in cucumber plants. In particular, on the 8th day of alkali stress, cucumbers treated with MT (SB-MT group) exhibited significantly higher levels of these substances than those subjected to alkali stress alone (SB group), although no significant differences were observed on the 4th day. These findings suggest that exogenous MT plays a crucial role in the synthesis of osmotic regulatory substances in cucumber under alkali stress.

### Exogenous MT regulates ROS detoxification in cucumber under alkali stress

**Fig. 5:**
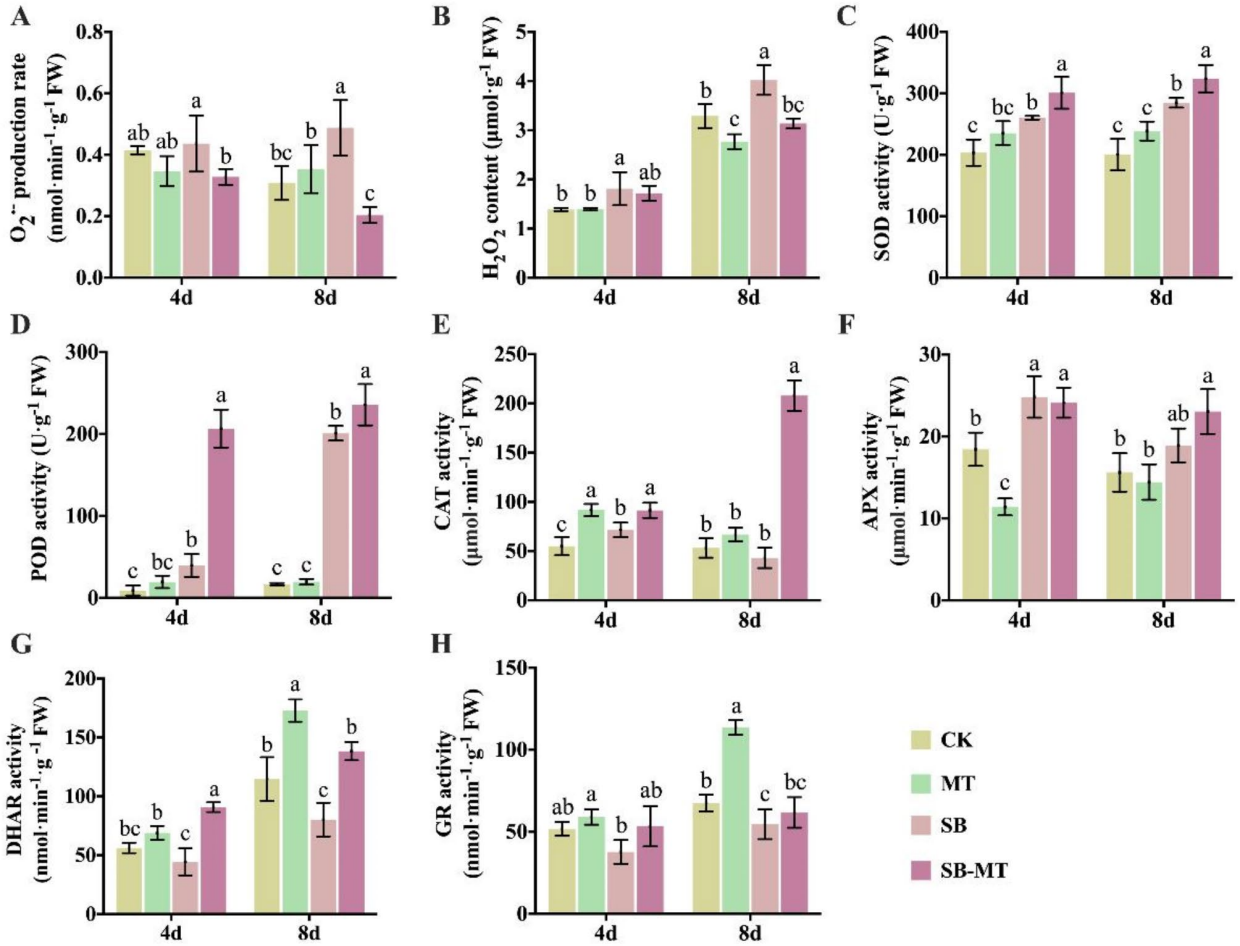
Effects of exogenous MT on ROS detoxification in cucumber seedlings exposed to alkali stress. (**A**–**B**) Reactive oxygen species (ROS) superoxide anion radical (O_2_.^−^) production rate and hydrogen peroxide (H_2_O_2_) content. (**C**–**H**) Antioxidant enzyme activities of cucumber leaves under NaHCO_3_ treatment. (**C**) Superoxide dismutase (SOD); (**D**) Peroxidase (POD); (**E**) Catalase (CAT); (**F**) Ascorbate peroxidase (APX); (**G**) Dehydroascorbate reductase (DHAR); (**H**) Glutathione reductase (GR). Values are means ± SD (*n* = 3). Different letters indicate significant differences (*p* < 0.05)

We further focused on the antioxidant regulatory pathway to analyze the mechanism by which MT regulates cucumber alkali stress resistance. Figure 5 illustrates that, when exposed to alkali stress, there was a notable increase in the accumulation of O_2_.^−^ (Fig. 5A) and H_2_O_2_ (Fig. 5B) in cucumber plants. However, the application of MT externally led to a significantly reduced production of these two substances. Furthermore, the activities of SOD (Fig. 5C), POD (Fig. 5D), and CAT (Fig. 5E) were markedly increased under alkali stress compared to those in the control group (CK), but were still significantly lower than those observed in the SB-MT group. While APX activity (Fig. 5F) was enhanced under alkali stress, there was no significant difference compared with that in the SB-MT group. The activities of DHAR (Fig. 5G) and GR (Fig. 5H) markedly declined after 8 d of alkali stress. In contrast, application of exogenous MT significantly increased DHAR activity under alkali stress; however, the enhancement of GR activity was not significant. Furthermore, under normal growth conditions, the activities of DHAR and GR increased with the growth of cucumber seedlings, and the exogenous addition of MT significantly enhanced these activities. These findings suggest that MT can bolster the antioxidant pathway in cucumber under alkali stress, thereby enhancing the tolerance of plants to such conditions.

### Exogenous MT regulates ion homeostasis in cucumber under alkali stress

**Fig. 6:**
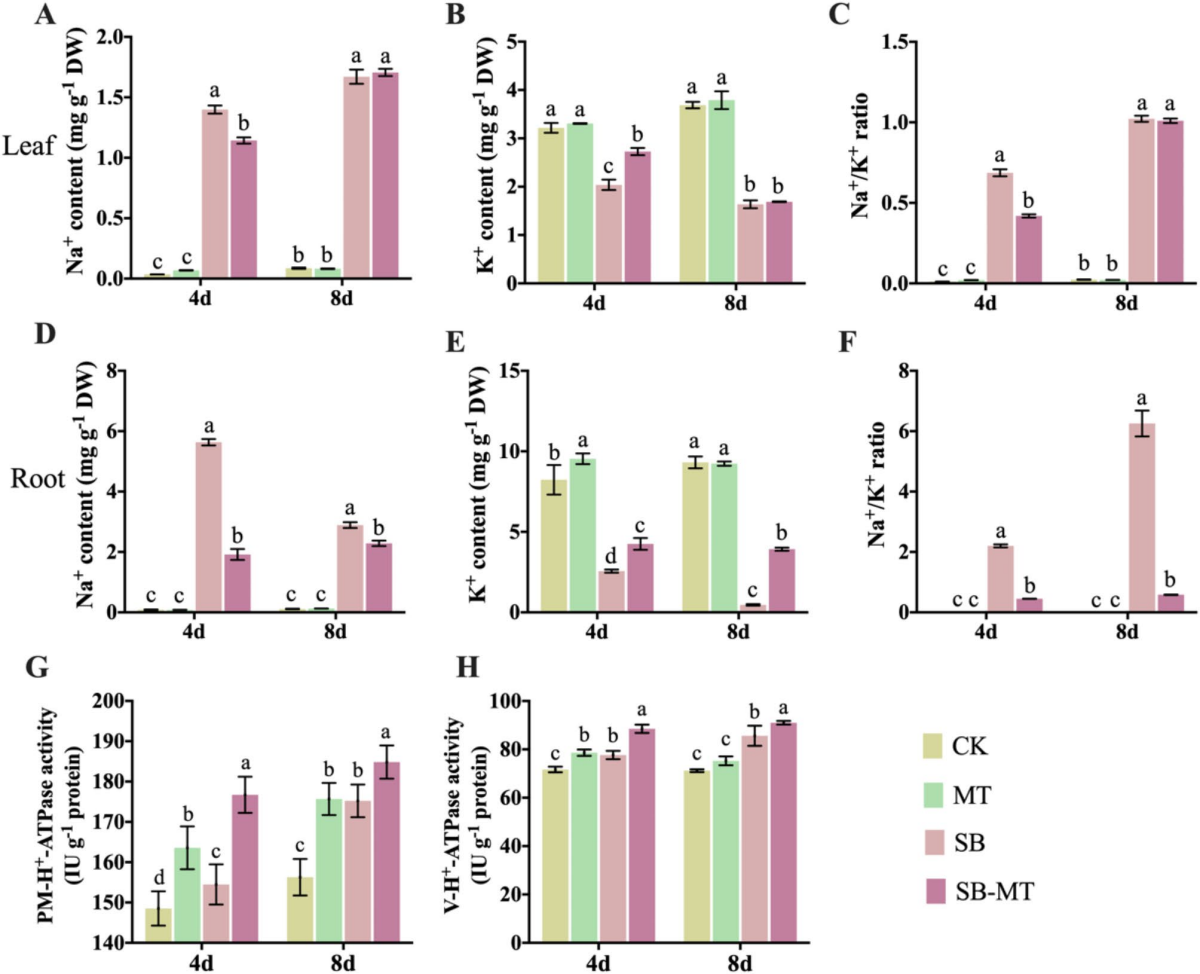
Effects of exogenous MT on ion balance regulation in cucumber seedlings exposed to alkali stress. (**A**–**C**) Levels of sodium ions (Na^+^; A), potassium ions (K^+^; B), and Na^+^/K^+^ ratio (**C**) in cucumber leaves from different experimental groups under NaHCO_3_ treatment. (**D**–**F**) Levels of Na^+^ (**D**), K^+^ (**E**), and Na^+^/K^+^ ratio (**F**) in cucumber roots from different experimental groups under NaHCO_3_ treatment. (**G**–**H**) Plasma membrane (P) H^+^-ATPase activity and vacuolar membrane (V) H^+^-ATPase activity in cucumber leaves from different experimental groups under NaHCO_3_ treatment. Values are means ± SD (*n* = 3). Different letters indicate significant differences (*p* < 0.05)

The ion balance regulatory pathway is crucial for plant resistance to alkali stress. Plants maintain an intracellular ion balance by increasing Na^+^ efflux and promoting K^+^ absorption to mitigate the damage caused by alkali stress. Figure 6 illustrates that, under normal growth conditions, cucumber leaves and roots exhibit low Na^+^ content (Fig. 6A and D) and high K^+^ content (Fig. 6B and E). The application of MT does not have a significant overall impact on Na^+^ and K^+^ levels, leading to an insignificant difference in the Na^+^/K^+^ ratio (Fig. 6C and F). However, when subjected to alkali stress, the Na^+^ content in cucumber leaves and roots increased notably, whereas the K^+^ content decreased significantly. The external application of MT (SB-MT group) led to a significant reduction in Na^+^ content and a notable increase in K^+^ content in cucumber leaves and roots, particularly on the 4th day of the treatment, resulting in a remarkable decrease in the Na^+^/K^+^ ratio. However, by the 8th day, no significant differences were observed in the Na^+^ and K^+^ content in the leaves. This may be attributed to the severe rotting of the roots in SB group seedlings, which compromised the plants’ functionality and their ability to absorb and transport Na^+^. Furthermore, the activities of PM-H^+^-ATPase (Fig. 6G) and V-H^+^-ATPase (Fig. 6H) in cucumber leaves increased substantially under alkali stress. Interestingly, the external application of MT not only boosted the activities of these enzymes in cucumbers under alkali stress, but also under normal conditions. This suggests that MT plays a basic role in regulating the ion balance pathways in cucumbers.

### Exogenous MT regulates stress-related gene expression in cucumber under alkali stress

**Fig. 7:**
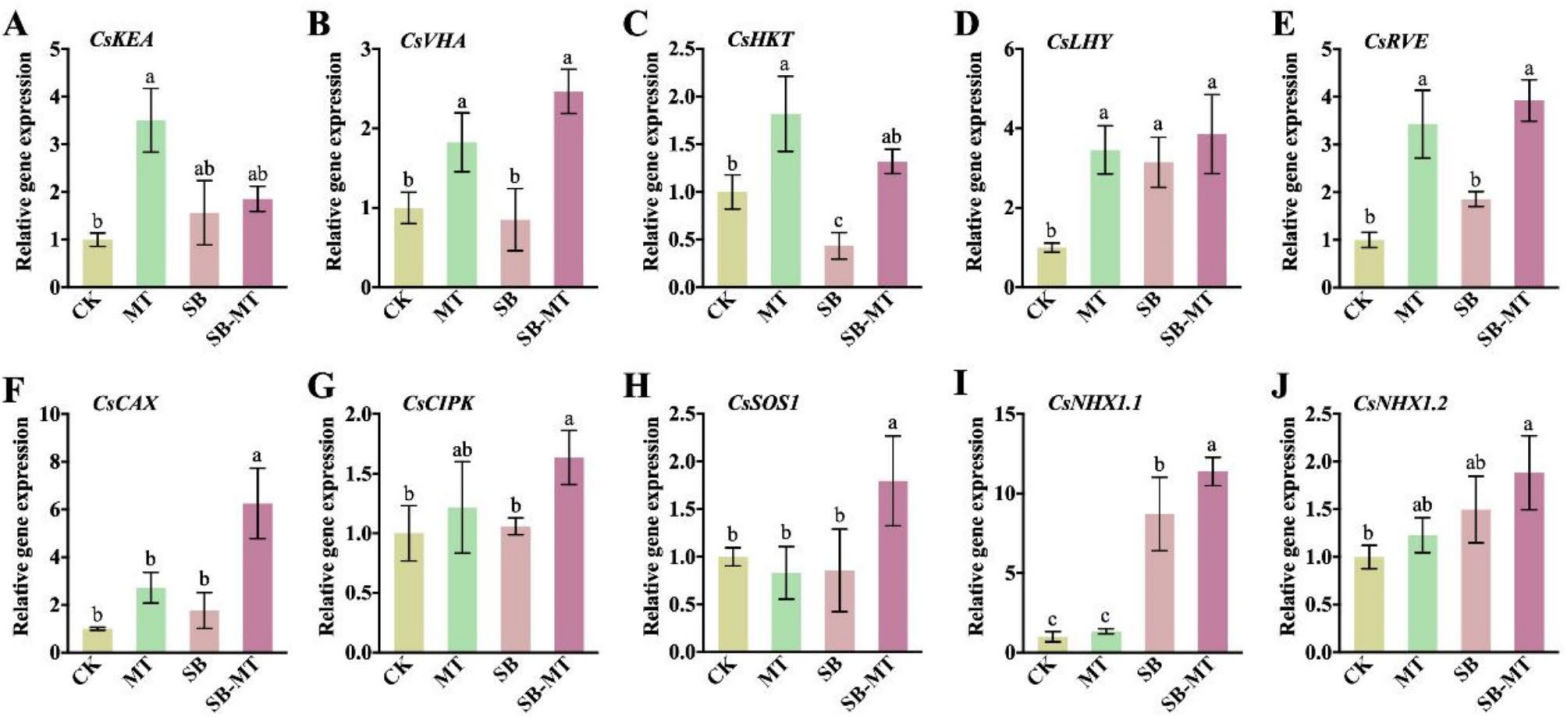
Effects of exogenous MT on stress-related gene expression in cucumber seedlings exposed to alkali stress. Cucumber leaves were collected on the 4th day of treatment to analyze the relative expression of stress-related genes. This included *CsKEA* (K^+^ efflux antiporter 3, Gene ID: *CsaV3_7G007280*) (**A**), *CsVHA* (V-type proton ATPase proteolipid subunit, Gene ID: *CsaV3_3G046610*) (**B**), *CsHKT* (high affinity K^+^ transporter, Gene ID: *CsaV3_1G032160*) (**C**), *CsLHY* (protein LHY-like isoform X2, Gene ID: *CsaV3_6G008360*) (**D**), *CsRVE* (protein REVEILLE 8-like isoform X3, Gene ID: *CsaV3_3G035450*) (**E**), *CsCAX* (Ca^2+^/H^+^ exchanger antiporter, Gene ID: *CsaV3_6G016610*) (**F**), *CsCIPK* (SOS2-like protein kinases, Gene ID: *CsaV3_UNG100750*) (**G**), *CsSOS1* (Na^+^/H^+^ exchanger 7, Gene ID: *CsaV3_5G017250*) (**H**), *CsNHX1.1* (Na^+^/H^+^ exchanger 1, Gene ID: *CsaV3_7G028690*) (**I**), and *CsNHX1.2* (Na^+^/H^+^ exchanger 1, Gene ID: *CsaV3_6G005700*) (**J**). Expression level in CK group was set as 1. The cucumber actin gene (*CsaV3_6G041900*) was used as an internal control, and each experiment was repeated three times. Different letters indicate significant differences (*p* < 0.05)

To further investigate the molecular mechanism underlying the enhancement of alkali tolerance in cucumber seedlings by exogenous MT, we analyzed the expression of salt-alkali stress-related genes *CsKEA* (Fig. 7A), *CsVHA* (Fig. 7B), *CsHKT* (Fig. 7C), *CsCAX* (Fig. 7F), *CsCIPK* (Fig. 7G), *CsSOS1* (Fig. 7H), *CsNHX1.1* (Fig. 7I), *CsNHX1.2* (Fig. 7J) as well as two circadian rhythm-related genes *CsLHY* (Fig. 7D) and *CsRVE* (Fig. 7E). The results revealed that the external application of MT under normal conditions led to an increased expression of *CsKEA*, *CsVHA*, *CsHKT*, *CsLHY*, and *CsRVE*, with significantly higher levels than those in the CK group. When subjected to alkali stress alone, the gene expression of *CsKEA*, *CsLHY*, *CsRVE*, *CsCAX*, *CsCIPK*, *CsNHX1.1*, and *CsNHX1.2* was upregulated compared to the CK group, but only *CsLHY* and *CsNHX1.1* showed a significant difference. Furthermore, *CsVHA*, *CsHKT* and *CsSOS1* were downregulated, and *CsHKT* showed a significant decrease. Compared to the SB group, the expression of *CsVHA*, *CsHKT*, *CsRVE*, *CsCAX*, *CsCIPK*, *CsSOS1* and *CsNHX1.1* was significantly increased in the SB-MT group. These findings indicate that exogenous MT can enhance the alkali tolerance of cucumbers by promoting the expression of stress-related genes under both normal and alkali stress conditions.

### Exogenous MT promotes the synthesis of endogenous MT in cucumber under alkali stress

**Fig. 8:**
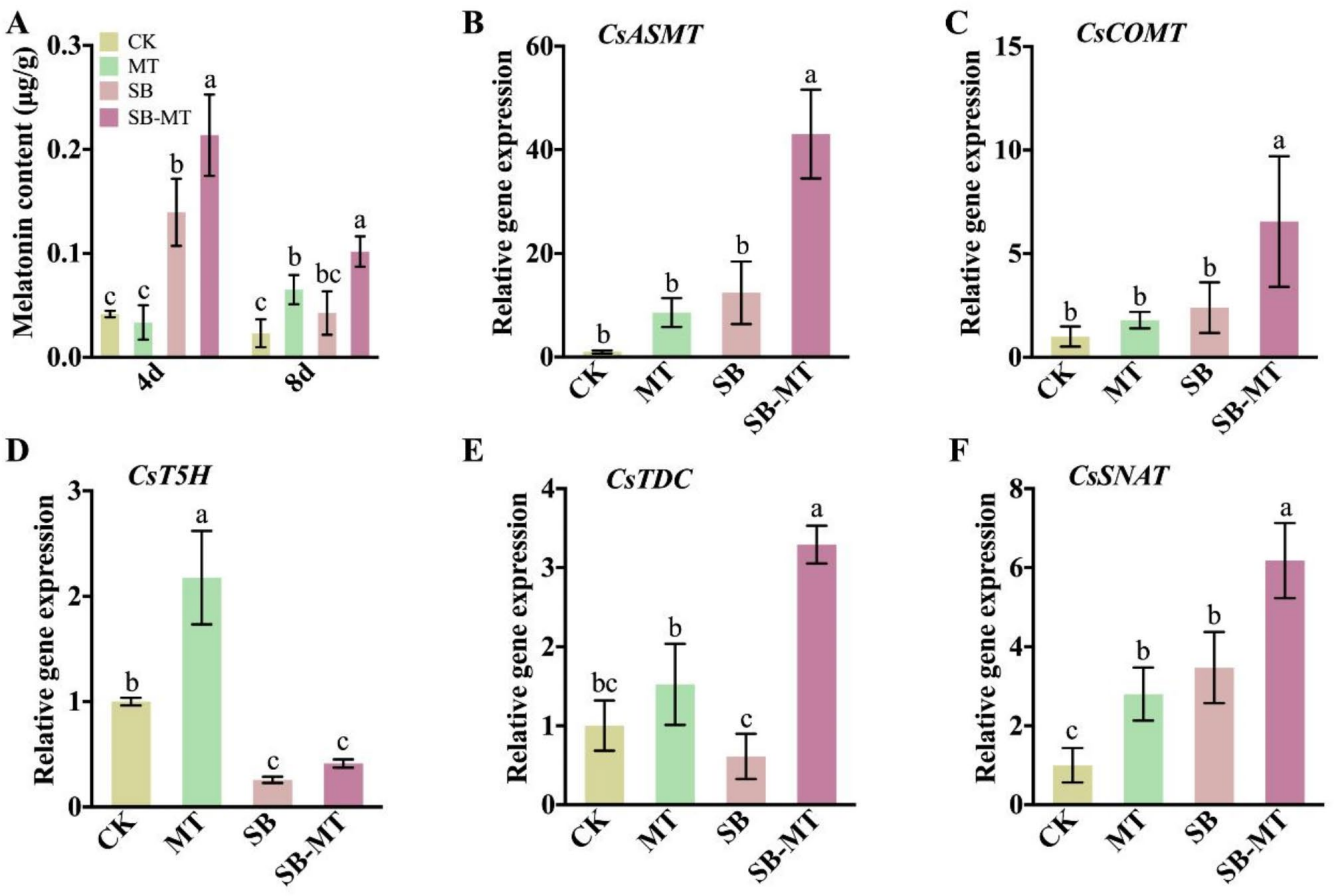
Effects of exogenous MT on the endogenous MT synthesis in cucumber seedlings exposed to alkali stress. Cucumber leaves were collected on the 4th and 8th days of treatment to quantify melatonin levels (**A**), and the gene expression involved in melatonin synthesis pathway, including *CsASMT* (Acetylserotonin O-methyltransferase, Gene ID: *CsaV3_7G003180*) (**B**), *CsCOMT* (Caffeic acid O-methyltransferase, Gene ID: *CsaV3_4G008590*) (**C**), *CsT5H* (Tryptamine 5-hydroxylase, Gene ID: *CsaV3_6G045890*) (**D**), *CsTDC* (Tryptophan decarboxylase, Gene ID: *CsaV3_3G028450*) (**E**), and *CsSNAT* (Serotonin N-acetyltransferase, Gene ID: *CsaV3_4G027340*) (**F**) were analyzed on the 4th day. Expression level in CK group was set as 1. The cucumber actin gene (*CsaV3_6G041900*) was used as an internal control, and each experiment was repeated three times. Different letters indicate significant differences (*p* < 0.05)

To investigate the role of exogenous MT in regulating endogenous MT synthesis in cucumbers under alkali stress, we analyzed both MT content and the expression of MT synthesis pathway-related genes. The results presented in Fig. 8A demonstrate that on the 4th day of the experiment, there was no significant difference in the MT content between the MT and CK groups. However, by the 8th day, the MT content in the MT group was notably higher than that in the CK group, suggesting that external MT application enhanced the synthesis of endogenous MT, albeit in a time-dependent manner. Furthermore, on the 4th day of treatment, the endogenous MT levels in the SB group were significantly higher than those in the CK group. Conversely, by the 8th day, endogenous MT levels in the SB group decreased significantly compared to the 4th day, with no significant difference observed when compared to the CK group. These findings indicate that exposure to alkali stress can stimulate endogenous MT synthesis in cucumber plants, whereas severe alkali stress can disrupt cucumber metabolism, leading to decreased endogenous MT levels. Moreover, the endogenous MT levels in the SB-MT group were significantly higher than those in the SB group on both the 4th and 8th days of treatment, suggesting that exogenous MT application under alkali stress conditions can boost endogenous MT synthesis, thereby improving the alkali tolerance of cucumber seedlings. On the 4th day of treatment, the relative expression levels of the MT synthesis-related genes, *CsASMT*, *CsCOMT*, *CsT5H*, *CsTDC*, and *CsSNAT* were higher in the MT group than in the CK group. Notably, *CsT5H* and *CsSNAT* exhibited significant differences. In the SB group, while *CsASMT*, *CsCOMT*, and *CsSNAT* showed higher expression levels than in the CK group, only *CsSNAT* displayed a significant difference. Conversely, *CsTDC* levels were lower than those in the CK group, and *CsT5H* levels were significantly lower. Furthermore, the relative expression levels of *CsASMT*, *CsCOMT*, *CsTDC*, and *CsSNAT* were significantly higher in the SB-MT group than in the SB group, with no significant difference in *CsT5H*. These findings suggest that exogenous MT enhances the expression of MT synthesis genes under normal growth conditions and alkali stress, ultimately leading to increased MT accumulation.

## Discussion

### Exogenous MT promotes seed germination and early seedling establishment in cucumber under normal growth conditions

Seed germination and seedling establishment are critical processes in the plant life cycle and contribute to the determination of crop quality and yield. Therefore, promoting seed germination and enhancing seedling vigor have emerged as the main objectives in crop breeding [54]. Although numerous studies have demonstrated that exogenous MT enhances seed germination and seedling growth under normal and stressed conditions [55, 56], comprehensive research on the regulation of exogenous MT during seed germination and subsequent seedling establishment under normal growth conditions remains insufficient, particularly in cucumber crops. It remains to be determined whether exogenous MT exerts similar effects at different developmental stages of cucumber seed germination and early seedling establishment and whether its effects vary with different concentrations.

Previous studies have demonstrated that, under normal growth conditions, the application of exogenous MT can enhance the germination of seeds from cotton (*Gossypium hirsutum* L.) [57], *Paeonia ostia* ‘Fengdan’ [58], and *Stevia rebaudiana* Bertoni [35], etc. However, the optimal concentration of MT differs significantly among species. Generally, low concentrations of MT are found to promote germination, whereas high concentrations tend to inhibit this process, which is consistent with our findings. In our study, we found that 5 µM MT enhanced cucumber seed germination, whereas higher concentrations (100, 200, and 400 µM) significantly inhibited the germination process under normal conditions (Fig. 1). Conversely, Zhang et al. [59] reported that melatonin concentrations of 0, 0.1, 1, 10, 100, and 500 µM did not have a significant direct effect on cucumber seed germination under normal conditions. However, MT was shown to effectively promoted cucumber seed germination under conditions of salt stress [59], low temperature [60], and water stress [29], with optimal promotion observed at concentrations of 1, 50, and 100 µM, respectively. Notebly, Lv et al. [28] demonstrated that exogenous MT inhibited seed germination in *Arabidopsis* by interacting with abscisic acid, gibberellin, and auxin under normal conditions. In their study, the authors selected MT concentrations of 0, 10, 100, 500, and 1000 µM for experiments. They found that low concentrations of MT (0, 10, and 100 µM) had no significant effect on seed germination, while higher concentrations (500 and 1000 µM) significantly inhibited germination. We propose that the observed phenomenon in Lv et al.’s study may be attributed to the use of high concentrations of MT. However, Lv et al. also confirmed that the MT biosynthesis enzyme gene mutants, *amst* and *comt*, exhibited higher seed germination rates. Conversely, the germination rate of *AMST* overexpression lines was significantly reduced. These findings are inconsistent with other studies. The discrepancies in these findings may be attributed to differences in species, the selection of MT concentrations, and the processing conditions employed. These factors also highlight the intricate complexity of the mechanisms in the MT-mediated seed germination. Researches indicate that MT facilitates seed germination under normal conditions by modulating the synthesis of plant hormones. Specifically, it enhances seed germination by decreasing abscisic acid (ABA) levels while simultaneously increasing gibberellin (GA_3_) levels. Furthermore, MT enhances the activity of $\:\alpha\:$-amylase, which catalyzes the conversion of starch into simple sugars, supplying the essential energy required for seed germination [58, 61]. However, there is currently a lack of comprehensive research analyzing the mechanisms by which MT regulates seed germination under normal growth conditions.

Early seedling establishment is critical for subsequent growth and yield. In the present study, low concentrations of MT, particularly 5 µM, significantly enhanced the growth of both the above-ground and root portions of post-germination seedlings. In contrast, high concentrations of MT (100 and 200 µM) markedly inhibited overall growth; interestingly, they promoted stem thickening and elongation (Fig. 2). The underlying regulatory mechanisms warrant further exploration. Zhao et al. [30] found that the endogenous MT content in the flower stems of various herbaceous peony varieties was positively correlated with the strength of these stems. They further elucidated that the primary regulatory mechanism by which MT enhances the strength of herbaceous peony flower stems involves an increase in lignin accumulation and the thickness of the secondary cell wall. Besides, we further applied MT (5 µM) externally at the one-leaf stage of the seedlings and found that it significantly promoted root growth; however, the effect on the aboveground parts was not significant (Fig. 3). These results demonstrate that exogenous MT exerts varying effects on cucumber growth at different developmental stages, with a more pronounced impact on root development compared to shoot growth, potentially due to its auxin-like functions [36]. Sarropoulou et al. [62] showed that low concentrations of MT promoted the root growth of commercial sweet cherry rootstocks, while high concentrations inhibited this growth, exhibiting a similar action to that of IAA. Yang et al. [31] further demonstrated that MT promotes the growth of the primary root in Arabidopsis in an IAA-dependent manner.

The above results indicate that the mechanisms by which exogenous MT-mediated seed germination and early seedling establishment are highly complex. In a previous study, we identified two members of the TDC gene family (key enzymes in the MT synthesis pathway) in the cucumber genome [63]. Both genes exhibited the highest expression levels in the seeds and roots, suggesting a potential role in MT-mediated seed germination and seedling growth, particularly root formation; however, the specific regulatory mechanisms involved require urgent elucidation.

### Exogenous MT enhances the alkaline stress tolerance of cucumber seedlings

To comprehensively investigate the role of exogenous MT in cucumbers, we further examined its effect on alkali tolerance. Our findings demonstrated that alkali stress significantly inhibited the growth of cucumber seedlings. However, the external application of MT mitigated the damage caused by this stress. Subsequently, we analyzed the alkali tolerance mechanisms of cucumbers mediated by exogenous MT, focusing on osmotic regulation (Fig. 4), reactive oxygen species scavenging (Fig. 5), ion balance (Fig. 6), expression of alkali stress-related genes (Fig. 7), and endogenous MT regulatory pathways (Fig. 8).

Under saline-alkali stress, the accumulation of salt-alkali ions around the rhizosphere of plants results in an increased soil osmotic pressure that exceeds the osmotic pressure of plant cells. This discrepancy reduces the water uptake capacity of plant root cells, leading to osmotic and physiological drought stress [7, 64]. Consequently, plants actively accumulate solutes in saline-alkali environments to maintain their intracellular water potential and enhance water uptake. Proline and soluble sugars are crucial osmoregulatory substances, and various studies have shown that their concentrations increase under stress conditions, thereby enhancing plant stress tolerance [13]. Zhu et al. [65, 66] demonstrated that the application of silicon enhances the accumulation of proline and soluble sugars under salt stress, which promotes root water uptake and improves the salt stress resistance of cucumbers, thereby highlighting the significance of osmotic adjustment in cucumber’s resistance to salt stress. In this study, we observed that the exogenous application of MT significantly promoted the production of osmoregulatory substances in cucumbers subjected to alkali stress (Fig. 4). These findings indicate that exogenous MT plays a role in regulating osmotic pressure in cucumbers under alkali stress by stimulating the synthesis of osmoregulatory substances, which stabilize cellular osmotic potential and mitigate the damage inflicted on cucumber seedlings by alkali stress.

Saline-alkali stress can lead to excessive accumulation of ROS in plants, resulting in oxidative stress that is detrimental to cellular metabolism and impede plant development [10]. In response to oxidative damage, plants typically activate ROS-scavenging systems, which include antioxidant enzymes such as SOD, POD, and APX, as well as antioxidants such as ascorbic acid, reduced glutathione, and flavonoids, to maintain ROS homeostasis [10, 67]. Mitigating oxidative damage is a crucial strategy that enables cucumbers to withstand salt stress [13, 68]. MT and its metabolites are recognized as effective antioxidants and free radical scavengers that can directly scavenge or enhance antioxidant enzymes activities to reduce the production of ROS, alleviate oxidative stress-induced damage, and enhance plant tolerance to stress [69]. Numerous studies have shown that the external application of MT improves the resistance of crops to salt stress by decreasing ROS levels, including apple [70], maize [71], pepper [72], and tea [73]. Additionally, MT and nitric oxide interact to modulate the expression of the SOD enzymes, enhancing the salt resistance of sunflowers [74]. In this study, we observed that the pretreatment with exogenous MT significantly mitigated the accumulation of O_2_^⋅−^ and H_2_O_2_. Concurrently, the exogenous application of MT markedly enhanced the activities of SOD, POD, CAT, and DHAR in cucumbers subjected to alkali stress, indicating that exogenous MT could bolster the antioxidant capacity of cucumber seedlings under such conditions (Fig. 5). Studies have shown that exogenous MT promotes the production of endogenous MT in plants under salt stress [75]. The application of exogenous MT considerably enhanced the expression of genes related to melatonin synthesis, resulting in elevated levels of endogenous MT in grapes [76]. Our results indicate that exogenous MT not only promotes the synthesis of endogenous MT under normal growth conditions but also under alkali stress. Notably, external application of MT significantly upregulated the expression of MT synthase genes, namely *CsASMT*, *CsCOMT*, *CsTDC*, and *CsSNAT*, under alkali stress (Fig. 8). Collectively, these results indicated that exogenous MT facilitates endogenous MT production in cucumbers exposed to alkali stress. Both exogenous and endogenous MT act as antioxidants and collaboratively regulate endogenous antioxidant defense systems to protect cells against oxidative stress.

In addition to inducing osmotic and oxidative stress, alkali stress can lead to ion toxicity and elevated pH [7]. PM-H^+^-ATPase plays a crucial role in plant resistance to salt-alkali stress. On the one hand, it provides a proton gradient for SOS1-mediated Na^+^ efflux, and on the other hand, it promotes protons efflux from the cytoplasm via ATP hydrolysis, thus maintaining pH balance inside and outside the cell [11, 15]. Our results indicate that the external application of MT significantly mitigates Na^+^ accumulation under alkali stress (Fig. 6). This effect may be attributed to the substantial increase in PM-H^+^-ATPase activity (Fig. 6) and the expression of the *CsCIPK* and *CsSOS1* genes (Fig. 7). The serine/threonine protein kinase SOS2, encoded by *CsCIPK*, activates the *CsSOS1*-encoded plasma membrane Na^+^/H^+^ antiporter, which promotes the efflux of Na^+^ relying on proton gradients generated by PM-H^+^-ATPase, thereby reducing Na^+^ accumulation [15]. Additionally, melatonin markedly enhances V-H^+^-ATPase activity (Fig. 6) and the expression of *CsVHA* and *CsNHX1.1* genes (Fig. 7). *CsVHA* encodes a subunit of the V-H^+^-ATPase. The *CsNHX1.1*-encoded tonoplast Na^+^/H^+^ antiporter NHX1 compartmentalizes excess Na^+^ in the vacuole under the proton gradient provided by V-H^+^-ATPase, thereby inhibiting the transport of excess Na^+^ to the aboveground and reducing damage to the aboveground [15, 16]. Besides, in our study, the external application of MT significantly enhanced K^+^ accumulation under alkali stress (Fig. 6). On one hand, MT alleviates the inhibition of K^+^ absorption caused by the excessive accumulation of Na^+^. On the other hand, MT markedly increases the expression level of *CsHKT* (Fig. 7), which encodes a high-affinity K^+^ transporter that facilitates K^+^ absorption under saline-alkali stress, thereby playing a crucial role in maintaining Na^+^ and K^+^ balance [17, 18]. The above results suggest that exogenous MT plays a crucial role in regulating Na^+^ and K^+^ homeostasis by modulating the expression of genes associated with ion homeostasis and proton pump activity, thereby reducing ion toxicity, and enhancing alkali stress tolerance in cucumber. Furthermore, alkali stress leads to the precipitation of Ca^2+^ in the soil, which impedes the absorption of Ca^2+^ by plants. Studies have demonstrated that Ca^2+^ function as second messenger molecules in plants, playing a crucial role in the regulation of plant stress resistance. The crosstalk between Ca^2+^ and MT is likely involved in initiating plant defense responses [77, 78]. In our study, we found that MT significantly enhanced the expression of *CsCAX*, a Ca^2+^/H^+^ exchanger antiporter, under alkali stress (Fig. 7). This suggests that MT may play a role in regulating Ca^2+^ signaling in cucumber plants under alkali stress.

In addition, plants can adapt to their environment and regulate biotic and abiotic stresses through internal circadian rhythms [79]. Studies have demonstrated that exogenous MT plays a role in regulating the plant circadian clock [47]. In this study, we found that exogenous MT significantly induced the expression of *CsRVE*, a core component of the circadian clock, under both normal and alkali stress conditions (Fig. 7). Research have shown that *CstMYB1R1*, a REVEILLE-8-like transcription factor, acts as a positive regulator of Crocus abiotic stress tolerance [80]. These results suggest that exogenous MT may play a role in regulating the circadian clock in cucumber plants subjected to alkali stress. This regulation appears to influence the expression of stress-related genes by modulating circadian rhythms, ultimately affecting alkali stress tolerance in cucumber seedlings. However, the precise regulatory mechanisms require further investigation.

**Fig. 9:**
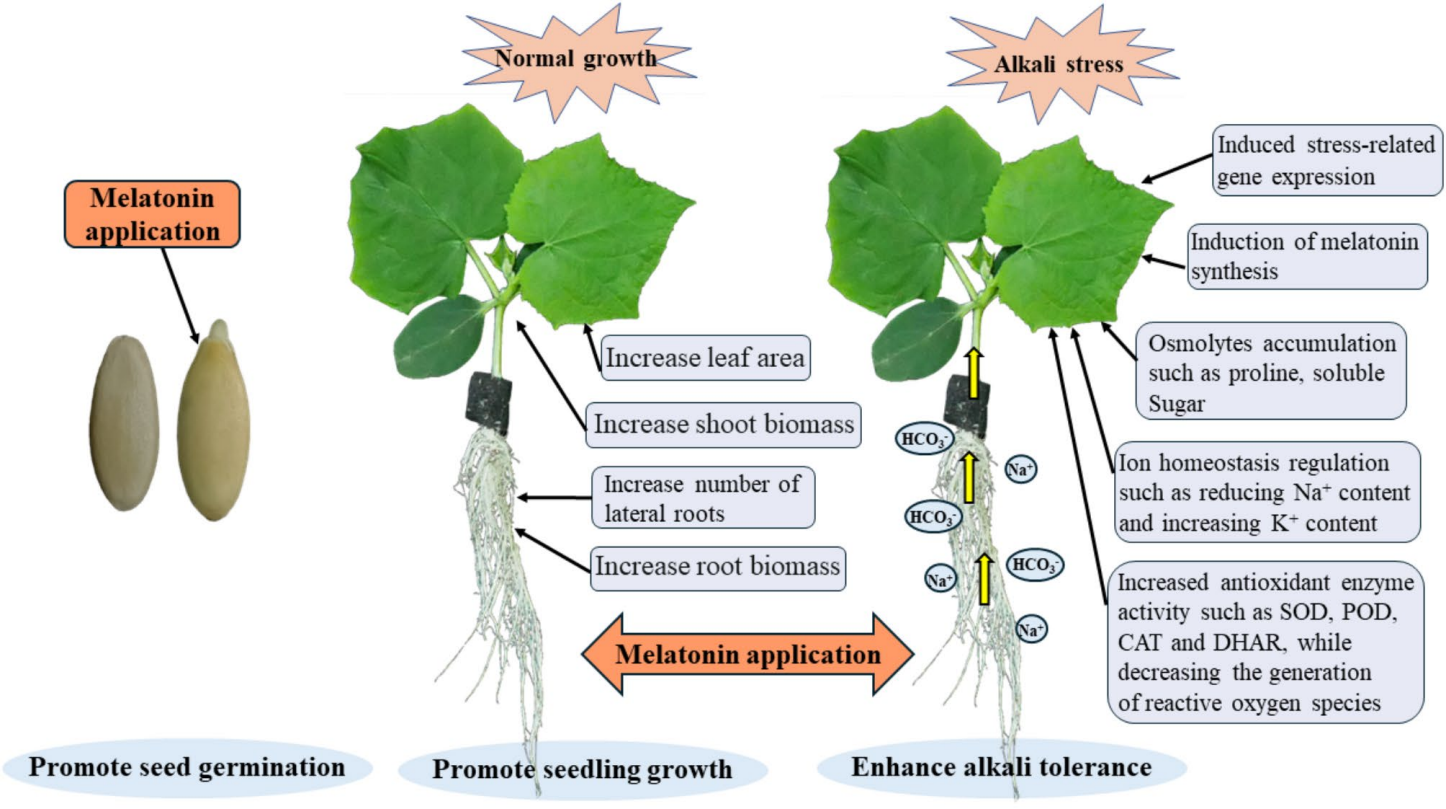
The working model illustrating the functions of exogenous MT on cucumber seed germination, seedling growth, and alkali stress regulation

In summary, our study conducted a thorough analysis of the effects of exogenous MT on cucumber seed germination, seedling establishment, and alkali tolerance. To effectively present our findings, we graphically illustrate the impact of MT on promoting the growth and stress resistance of cucumber seedlings through external application (Fig. 9). However, the specific regulatory mechanisms underlying these effects require further investigation.

## Electronic supplementary material

Below is the link to the electronic supplementary material.

Supplementary Material 1: Supplementary Table 1: Primers used for qRT-PCR analysis.

Supplementary Material 2: Supplementary Fig.1: Screening of NaHCO3 concentration for alkali stress treatment in cucumber seedlings.

## Data Availability

Data is provided within the manuscript or supplementary information files.
