# Supplementary material for: Multiple functions of exogenous melatonin in cucumber seed germination, seedling establishment, and alkali stress resistance
Source: BMC Plant Biol. 2025 Mar 19;25:359. doi: 10.1186/s12870-025-06359-3 (PMC11921661; doi:10.1186/s12870-025-06359-3)
Supplement: Supplementary file 1 — Supplementary Material 1: Supplementary Table 1: Primers used for qRT-PCR analysis. [file 12870_2025_6359_MOESM1_ESM.docx]

Supplementary Fig.1 illustrates that under the treatment of 75 mM NaHCO_3_, the cucumber seedlings exhibited nearly halted growth, accompanied by lodging and mortality of all seedlings. In contrast, seedlings subjected to 50 mM NaHCO_3_ experienced significant growth inhibition, with approximately half of the seedlings collapsing and dying. Meanwhile, the treatment with 30 mM NaHCO_3_ led to inhibited growth, however, the effect was not statistically significant. Consequently, we selected a NaHCO_3_ concentration of 50 mM for subsequent alkali stress treatments. In addition, in this preliminary experiment, the selected seedling stage was relatively small, resulting in plant mortality occurring on the third day of treatment. To achieve improved treatment effects, we decided to initiate the treatment at a slightly older seedling stage, that is, one leaf and one heart stage.


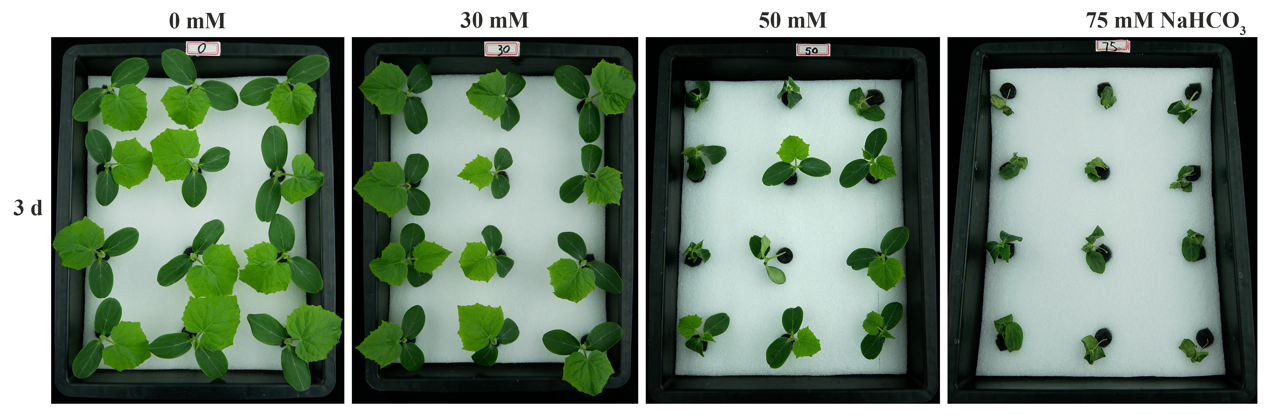


Supplementary Fig.1 Screening of NaHCO_3_ concentration for alkali stress treatment in cucumber seedlings. Once the cotyledons had half-unfolded, cucumber seedlings were transferred to a 1/4 Hoagland nutrient solution for cultivation. Upon the emergence of the first true leaf, the seedlings were subjected to NaHCO_3_ treatment at various concentrations (0, 30, 50, and 75 mM). The photographs were taken on the third day of treatment.
