# Supplementary figures and images for: Multiple functions of exogenous melatonin in cucumber seed germination, seedling establishment, and alkali stress resistance
Source: BMC Plant Biol. 2025 Mar 19;25:359. doi: 10.1186/s12870-025-06359-3 (PMC11921661; doi:10.1186/s12870-025-06359-3)

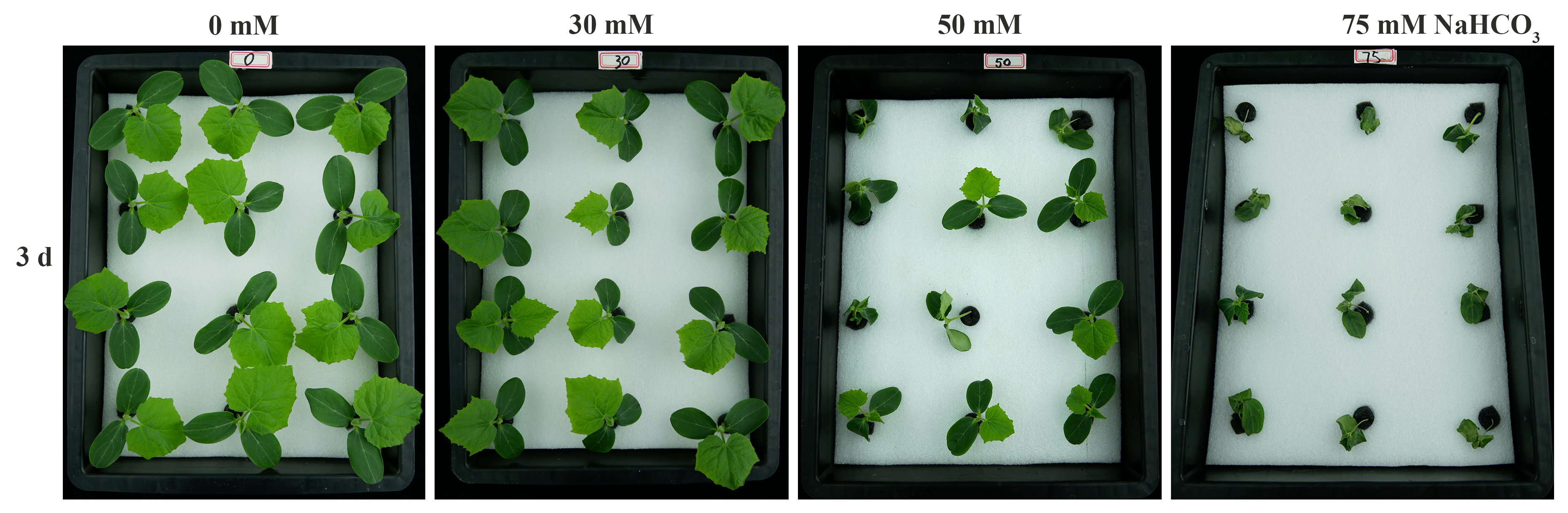

Supplement: Supplementary file 2 — Supplementary Material 2: Supplementary Fig.1: Screening of NaHCO3 concentration for alkali stress treatment in cucumber seedlings. [file 12870_2025_6359_MOESM2_ESM.tiff]
